# A scoping review of the toxicity and health impact of IQOS

**DOI:** 10.18332/tid/188867

**Published:** 2024-06-03

**Authors:** Sarah Ghazi, Min-Ae Song, Ahmad El-Hellani

**Affiliations:** 1 Division of Environmental Health Sciences, College of Public Health, The Ohio State University, Columbus OH, United States; 2 Center for Tobacco Research, The Ohio State University Comprehensive Cancer Center, Columbus OH, United States

**Keywords:** heated tobacco products, IQOS, toxicity, health effects

## Abstract

This work aims to summarize the current evidence on the toxicity and health impact of IQOS, taking into consideration the data source. On 1 June 2022, we searched PubMed, Web of Science, and Scopus databases using the terms: ‘heated tobacco product’, ‘heat-not-burn’, ‘IQOS’, and ‘tobacco heating system’. The search was time-restricted to update a previous search conducted on 8 November 2021, on IQOS data from 2010–2021. The data source [independent, Philip Morris International (PMI), or other manufacturers] was retrieved from relevant sections of each publication. Publications were categorized into two general categories: 1) Toxicity assessments included in vitro, in vivo, and systems toxicology studies; and 2) The impact on human health included clinical studies assessing biomarkers of exposure and biomarkers of health effects. Generally, independent studies used classical in vitro and in vivo approaches, but PMI studies combined these with modeling of gene expression (i.e. systems toxicology). Toxicity assessment and health impact studies covered pulmonary, cardiovascular, and other systemic toxicity. PMI studies overall showed reduced toxicity and health risks of IQOS compared to cigarettes, but independent data did not always conform with this conclusion. This review highlights some discrepancies in IQOS risk assessment regarding methods, depth, and breadth of data collection, as well as conclusions based on the data source.

## INTRODUCTION

Smoking cigarettes remains at alarmingly high rates worldwide (1.18 billion regular smokers) and is responsible for the annual death of 7 million casualties^1^. Efforts to curb this epidemic continue growing, including tobacco control policies, information campaigns, cessation care, and harm reduction approaches. During the last two decades, many nicotine and tobacco products have been introduced with reduced exposure and risk claims^2,3^. These alternative products with harm reduction potential include oral nicotine pouches, electronic cigarettes (ECs), and heated tobacco products (HTPs). An HTP that has gained global attention and rapid market expansion is IQOS, a product by Philip Morris International (PMI)^4^. IQOS was introduced into test markets in Japan and Italy in 2014, and within six years, its sales have expanded to over 60 countries^5^. IQOS relies on heating reconstituted tobacco at a temperature well below the temperatures measured in combustible cigarettes^4^. Recently, PMI secured a ‘modified exposure’ order from the US FDA based on a comprehensive modified risk tobacco product (MRTP) application^6^. However, the FDA found that PMI’s current data do not demonstrate that IQOS, as used by consumers, will significantly decrease the risk of tobacco-induced diseases for individuals or harm to the population^7^.

Nevertheless, several independent reports criticized the PMI data presented to the FDA^8^. For example, one report criticized the population health impact model used by PMI to justify that IQOS would benefit the individual and public health, and argued that this model excludes morbidity and underestimates mortality related to IQOS use in the population^9^. Also, independent researchers examined PMI data and found that claims of reduced exposure and risk are unsupported by the data^10-12^. Moreover, some independent researchers have encouraged policymakers to consider independent evidence before authorizing the marketing of IQOS and similar products that may harm public health^11,13^. Also, some health professional societies recommended that the toxicity of newly introduced tobacco products like IQOS should not be compared to combustible cigarettes but to no tobacco product use situations, i.e. focusing on the absolute, not relative toxicity^14^.

In this article, we conduct a literature review to assess the data on IQOS toxicity and health impact published by PMI-sponsored research (affiliated authors or funded studies) and independent research. Data from *in vitro*, *in vivo*, and systems toxicology studies were extracted to assess IQOS toxicity. The systems toxicology approach integrates multi-level biological data to comprehensively understand systemic molecular and functional changes from an omics-based method using computational modeling to extrapolate classical toxicology findings to risk assessment^15,16^. In addition, clinical studies were assessed for biomarkers of exposure and health effects of IQOS. This review aims to compare the cumulative evidence on the toxicity and health effects of IQOS from all data sources, including independent and tobacco industry-sponsored research while highlighting the methodological differences and conclusions among the studies listed.

**Figure 1:**
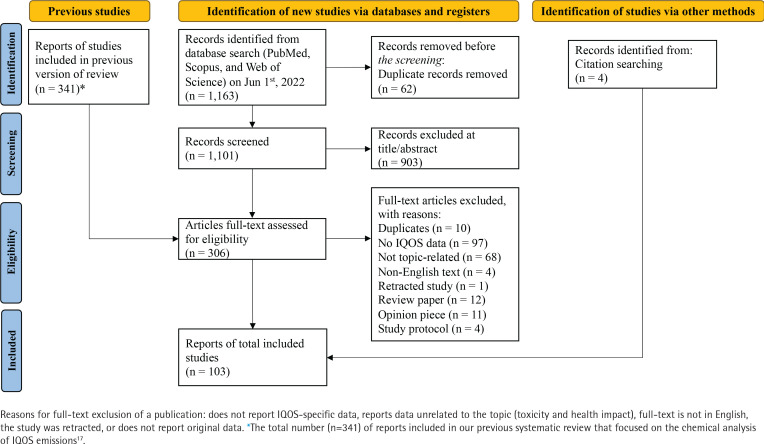
A flow chart diagram of the scoping review about the toxicity and health impact of IQOS with data from 2010–2021

We previously reported a systematic review on IQOS conducted on 8 November 2021, on Web of Science, PubMed, and Scopus using the terms ‘heated tobacco product’, ‘heat-not-burn’, ‘IQOS’, and ‘tobacco heating system’^17^. For the current scoping review, we looked at articles that assessed IQOS toxicity and health effects from our previous search. We also included more recent articles using the same search terms and methodology (up to 1 June 2022). Only reports written in English were included. A publication was excluded if it did not report IQOS-specific data, reported data unrelated to the topic (toxicity and health impact), or if the study was retracted or did not report original data. Figure 1 summarizes the selection process.

We extracted information on the data source [independent, PMI, or other heated tobacco product (HTP) manufacturers] from each publication’s author affiliation, conflict of interest, and/or study funding sections. Publications were categorized into two types of assessments: 1) toxicity, and 2) impact on human health. Toxicity assessments included *in vitro*, *in vivo*, and systems toxicology studies. The impact on the human health category included clinical studies assessing biomarkers of exposure and biomarkers of health effects (Figure 1).

## DEVELOPMENTS

**Figure 2:**
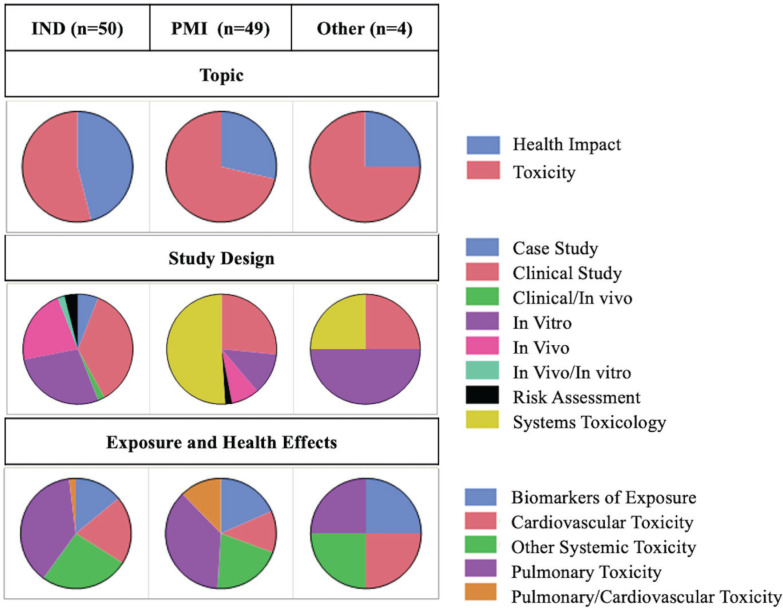
Categorization of publications based on the topic, study design, and exposure/health effects from independent research (IND), PMI, and other HTP manufacturers (Other) of the scoping review about the toxicity and health impact of IQOS, 2010–2021

Figure 2 shows the categorization of publications based on their topic, study design, and exposure/health effects, showing the distribution based on the data source. Only publications that reported original data were included (n=103) (Supplementary file Table S1). PMI data are presented first in each section below, followed by independent and competing manufacturers’ data (Figure 2).

### Toxicity assessment

Sixty-five toxicity assessment studies were classified based on their study designs (i.e. *in vitro*, *in vivo*, and systems toxicology studies). Then, they were subcategorized by research focus (i.e. pulmonary, cardiovascular, and other systemic toxicity).

*In vitro studies*

Pulmonary toxicity

PMI reported a combined 3D lung and liver tissue on a chip study showing that IQOS did not affect cytochrome P450 activity in both tissues^18^. Two other studies showed that after one week of exposure, total particulate matter (TPM) from IQOS had 20 times less effect on mitochondrial function in human bronchial epithelial cells compared to cigarette smoke (CS) exposure^19^. At prolonged exposure of 12 weeks, markers of cellular adaptation were observed^20^.

Several independent studies assessed pulmonary toxicity using *in vitro* methods. In a study of primary rat alveolar epithelial cells, IQOS exposure induced oxidative stress at 6 h. The authors concluded that this may lead to oxidative stress-related diseases like chronic obstructive pulmonary disease (COPD) and idiopathic pulmonary fibrosis (IPF) in humans^21^. Another study using an air-liquid interface (ALI) to assess the cytotoxic effects on human bronchial epithelial cells, showed that IQOS exposure induced higher cytotoxicity (reduced metabolic activity) than e-cigarettes or air controls but lower than combustible cigarettes^22^. While a study found that IQOS was less cytotoxic than CS to human lung epithelial cell line (A549) (90–95% estimated reduction in cytotoxicity), both products yielded reduced levels of glutathione (antioxidant) and increased carbonylation of proteins (markers of chronic lung diseases)^23^. A study of human bronchial epithelial cells (Beas-2B) and primary human airway smooth muscle cells found cytotoxicity to both cell types by IQOS, similar to CS and e-cigarettes^24,25^. A comprehensive study assessed the cytotoxic impact of IQOS gas phase, particle phase, and whole smoke emissions in comparison to Marlboro Red cigarettes on different types of human pulmonary cells [A549 and BEAS-2B cell lines, normal human bronchial epithelial cell (NHBE) cultures from different donors, normal human lung fibroblasts (NHLF), and human embryonic stem cells). The study reported that IQOS smoke (gas phase, particulate phase, or whole smoke] affected critical cellular functions and was equally cytotoxic to CS for several cell types, especially at high levels of exposure. This study showed that less cleaning of IQOS devices increased cytotoxicity^26^.

Cardiovascular toxicity

A study by PMI researchers showed that IQOS exposure had 18 times fewer inhibitory effects than CS on chemotaxis and trans-endothelial migration of human coronary arterial endothelial cells as a marker of cardiovascular health^27^. However, an independent study of the cytotoxicity of IQOS smoke on human vascular endothelial cells compared to cigarettes and other HTPs showed induced mitochondrial activity. IQOS decreased nitric oxide (NO) production, similar to other HTPs (e.g. Glo), but with lower effects than CS^28^. Similarly, IQOS and e-cigarette exposure were less cytotoxic than CS, less impacted endothelial wound healing of lab-simulated tissue injury, and reduced cellular stress response and inflammatory processes^29^.

Other systemic toxicity

A PMI study found that IQOS does not inhibit monoamine oxidase, which are enzymes suggested to be involved in smoking addiction, due to the reduced emission of possible monoamine oxidase inhibitors like acetaldehyde and 2-naphthylamine^30^. Another PMI study on human premolars showed that IQOS had minimal effects on teeth discoloration^31^. A study from a competing HTP manufacturer utilized a metabolomics assay to compare the developmental toxicity of IQOS, CS, and e-cigarettes with and without nicotine on human pluripotent stem cells. The data showed that IQOS crossed the developmental toxicity threshold at five times higher concentration than CS, unlike e-cigarettes that did not cross the threshold at maximum tested concentrations^32^.

Eight independent studies assessed the systemic toxicity of IQOS exposure or its effects on organs other than the pulmonary and cardiovascular systems. A study compared the effects caused by exposure to IQOS and CS on T lymphocytes’ oxidative balance and inflammatory parameters. While IQOS had smaller effects on T cell responses than CS exposure, IQOS smoke and CS impaired T cell proliferation, leading to cell death and decreased interleukin-2 (IL-2) secretion^33^. The effect of CS, IQOS, and e-cigarette aerosol extracts on the viability and differentiation of pre-adipocytes to beige adipocytes as a probe of the development of metabolic disorders was assessed, and only CS yielded detrimental effects^34^. In a study on the viability and function of human osteoprogenitors and mesenchymal cells, IQOS had significantly less toxicity in bone cells than CS^35^. However, another group reported a conflict finding, showing that IQOS exposure impairs preosteoblast cell viability and osteoblastic differentiation to a comparable extent as CS exposure^36^. A study found induced cell death and activated ferroptosis in a concentration and time-dependent manner in human corneal epithelial cell lines by exposure to IQOS or CS^37^. Another study found that IQOS can affect orbitopathy differently than CS^38^. The effect of IQOS exposure on teeth discoloration showed less impact than CS on artificial teeth color^39^, and IQOS was not cytotoxic on human keratinocytes and gingival fibroblasts (in the mouth gum)^40^.

*In vivo studies*

Pulmonary toxicity

A PMI study on chronic exposure of A/J Mice for 18 months to IQOS smoke showed that IQOS significantly reduced toxicity and carcinogenicity on red blood cell profile, liver function, lung inflammation, emphysematous, and histopathological changes compared to CS in respiratory tract organs^41^. Another 8-month exposure study showed that IQOS exposure caused hypermethylation of gene regulatory regions (i.e. promoters and enhancers) in both lung and liver tissues extracted from exposed mice (3 h/day, five days/week, for eight months), but the impact was smaller when compared to CS^42^.

In contrast, an independent study of the acute response of mice to IQOS exposure (1–2 days) showed a significant increase in oxidative stress and total lung glutathione, similar to the response after CS exposure^43^. Another study showed that compared to air-exposed controls, IQOS-exposed mice (1–4 days) had significantly decreased concentrations of reduced glutathione and increased percentage of oxidized glutathione in lung tissues, both markers of oxidative stress^44^. However, another study of mice exposure to IQOS emissions for 6 h/day for seven days did not find evidence of oxidative stress, measured by ROS, but found increased several proinflammatory mediators, including IL-1β and IL-6. This study showed that compared to e-cigarettes and CS, IQOS exposure was associated with lower lung injury^45^. A longer exposure study (5 h/day for two weeks) showed that both IQOS and CS exposure induced epithelial cell damage [higher levels of albumin in bronchioalveolar lavage (BAL)] compared to unexposed mice, yet a lower extent for IQOS. Although the accumulation of neutrophils, macrophages, and T cells in the lungs was lower in IQOS-exposed than in CS-exposed mice, the levels of proinflammatory cytokines and chemokines were similar in both groups^46^.

More independent data were reported on IQOS exposure compared to CS. A 1-month exposure study investigated the impact of IQOS on rat ultrastructural lung airways and found that IQOS exposure led to a severe remodeling of smaller and larger airways, increased tissue ROS, and promoted oxidative DNA damage; all factors are considered to increase lung cancer risk^47^. A recent study of mice exposed to IQOS aerosol for six months observed increased markers for pulmonary emphysema similar to those in CS exposure, indicating that IQOS is not completely safe. The authors found elevated levels of neutrophils and lymphocytes in the BAL fluid and upregulated genes involved in apoptosis-related pathways in IQOS-exposed mice^48^. A study that assessed the impact of long-term IQOS exposure (24 weeks) showed that IQOS exposure resulted in significantly reduced weight and lung function, higher inflammation, and higher oxidative stress compared to controls, and equivalent to CS exposure impact. The authors concluded that long-term exposure to IQOS could be detrimental to pulmonary health^49^.

Cardiovascular toxicity

Data from PMI on *in vivo* cardiovascular toxicity will be discussed in the systems toxicology section. Only two independent studies could be listed under *in vivo* cardiovascular toxicity. A study to determine the impact of IQOS exposure on vascular endothelial function in rats showed that exposure to emissions from a single IQOS Heatstick exerted similar impairment in arterial flow-mediated dilation as CS^50^. Another study found that all tobacco products, including IQOS and e-cigarettes, impair flow-mediated dilation in rats after a single exposure session^51^.

Other systemic toxicity

A meta-analysis of four *in vivo* studies conducted by PMI researchers assessed the impact of IQOS on the activity of the cytochrome P450 1 A2 (CYP1A2) enzyme responsible for the metabolism of harmful xenobiotics like amines. The results showed that switching the animals to IQOS caused the same effect as cessation of exposure to CS in terms of downregulating CYP1A2 activity to normal levels. The same observation was confirmed in four clinical studies (see below)^52^. Another PMI study showed that IQOS and CS have minimal impact on the intestinal microbiome in mice after six months of exposure^53^.

An independent study found higher expressions of metallothionein (scavengers of ROS and metals and associated with immune diseases and cancers) in the cells of the lungs and liver from mice exposed to CS but not to IQOS smoke^33^. A report examining PMI data on Sprague Dawley rats exposed to IQOS smoke or CS for 90 days observed increased markers of acute hepatotoxicity, including liver weight and alanine aminotransferase, in the IQOS-exposed group^54^. While a study showed aggravated arthritis symptoms in CS exposure only, IQOS and CS exposures affected lymphoid tissue cellularity and proliferation of splenocytes in mice during arthritis development^55^. Another study found that IQOS exposure impairs bone fracture healing to a similar extent when compared to CS-exposed mice^36^. A study of the impact of prenatal exposure to IQOS on testicular function showed more delayed sexual maturation and impaired spermatogenesis in male offspring compared to those in CS-exposed mice^56^.

*Systems toxicology*

No independent studies using systems toxicology were reported. PMI studies that used systems toxicology based on *in vitro* experiments will be summarized first. A PMI 3-day IQOS exposure study on human gingival epithelial organotypic cultures showed minor histopathological alterations, minimal cytotoxicity, and limited proinflammatory mediator alterations. The subsequent multi-omics analysis showed that IQOS induced about 79% lower biological impact when compared to CS in terms of alterations of genes related to oxidative stress, xenobiotic metabolism, and inflammation^57^. Another study on human organotypic oral epithelial cultures showed that IQOS, compared to CS, yielded less cytotoxicity (significant after 48 h post-exposure), secretion of proinflammatory mediators, and gene expression perturbations related to apoptosis, necroptosis, senescence, xenobiotic metabolism, and oxidative stress^58^. A study of 3D organotypic nasal epithelial culture showed that the impact of IQOS was substantially lower than CS in terms of cytotoxicity, tissue morphology, proinflammatory mediators, ciliary function, transcriptome perturbations, and miRNA expression profiles^59^. Regarding target organ effects, IQOS emitted much lower levels of harmful and potentially harmful constituents (HPHCs), induced lower cytotoxicity on normal primary human bronchial epithelial cells, and exerted lower overall biological impact (3 to 15 times lower than CS) as induced from systems toxicology analysis^60^. A long-term exposure study of IQOS (12 weeks) reported 20 times less toxicity on human bronchial epithelial cells regarding oxidative stress, DNA damage, and epithelial-to-mesenchymal transition (a marker of carcinogenesis)^61^. Similarly, IQOS elicited lower toxicity in all aspects than CS on lung epithelial cells and induced only 7.6% of the CS computationally estimated perturbation of gene expression^62^. Thus, a systems toxicology meta-analysis concluded that IQOS has reduced and more transient effects than CS on buccal, nasal, and bronchial epithelial cells regarding xenobiotic metabolism, oxidative stress, and inflammatory responses^63^. A study on small airway organotypic cells revealed that IQOS exposure induced lower cytotoxicity, lower secretion of proinflammatory mediators, and fewer transient perturbations in gene expression than CS exposure^64^. A recent study assessed 24-hour exposure of young and aged human aortic smooth muscle cells to IQOS and CS and showed no significant effect of IQOS on both cell groups in terms of cell proliferation, functional and molecular endpoints, and gene expression^65^. Another study assessing vascular pathomechanisms indicated a 10 to 20-fold lower effect of IQOS compared to CS on the adhesion of monocytic cells on human coronary arterial endothelial cells (a surrogate of atherogenesis)^66^.

An *in vivo* study showed low to absent effects of IQOS exposure on the inflammatory and oxidative stress response, immune response, and lipid and protein surfactant alterations in the lungs of mice after six months of exposure^67^. Another study showed that longer chronic exposure (18 months) to IQOS indicated lower toxic effects than CS on respiratory tract histology, lung inflammation, emphysematous changes, oxidative stress responses, and xenobiotic metabolism^68^. A 90-day nose-only inhalation exposure showed that IQOS had less impact than CS on body weight, hyperplasia and squamous metaplasia in the upper airway, lung inflammation, and overall biological impact (assessed by transcriptomic analysis). However, similar toxic effects between IQOS and CS were found on leukocyte counts in blood, cholesterol, glucose, liver-related enzyme activity, and weights for various organs and glands. The latter observation was attributed to the animals’ nicotine intake and experimental stress^69^. A similar study found the same reduction in toxicity when menthol-flavored IQOS was compared to mentholated reference cigarettes^70^. Follow-up systems toxicology studies showed that after 90 days of exposure, IQOS exposure, unlike CS exposure, did not lead to global miRNA downregulation while upregulating inflammation-related miRNA^71^ and menthol IQOS has minimal effect on lung proteomes and lipidomes^72^. Another study showed that ceasing mice’s exposure to CS, switching mice to IQOS after two months, or IQOS exposure for eight months, showed a similar reduced impact on lung lipids and lipid-related proteins, including surfactant lipids and proteins^73^.

IQOS’s impact on the cardiovascular system was also assessed. A study showed that mice exposure to IQOS emissions yielded no significant effect on cholesterol and low-density lipoprotein but increased high-density lipoprotein compared to controls but at a much lower impact than CS, and led to reduced development of atherosclerotic plaques. IQOS exposure also impacted lung volume and function less, inflammation and inflammatory cell infiltration in lung tissues, and less lung injury and emphysematous changes. These reduced effects were also reflected in the absence of IQOS-induced heart, lung, and thoracic aorta gene perturbations^74,75^. A follow-up study showed that IQOS exposure did not affect heart weight, left ventricular structure, atherosclerosis progression, heart function, and gene expression related to atherosclerosis and cardiovascular diseases^76^. Another study showed that eight months of mice exposure to IQOS did not induce atherosclerotic progression (aortic plaque formation), altered lipid profiles, upper airway epithelial hyperplasia and metaplasia, lung inflammation, and progressive emphysematous changes as CS exposure did. Lung morphometry and transcriptomics modeling corroborated the experimental results^77^. PMI researchers also used systems toxicology to evaluate the hepatotoxicity of 8-month IQOS exposure in mice. They showed that IQOS, unlike CS, did not induce alterations in lipid metabolism, xenobiotic metabolism, and iron homeostasis that could be linked to oxidative stress and liver function impairment^78^.

A study by a competing HTP manufacturer assessed the transcriptomic perturbations in 3D nasal airway cells acutely exposed to IQOS emissions compared to Glo and CS. The data showed altered expression levels of genes after exposure to IQOS and Glo (115 genes and 2 genes, respectively) compared to thousands of perturbations with CS exposure (2809 genes). In a separate analysis of cytokines, they did not find inflammation effects^79^.

### Health impact

*Biomarkers of exposure*

A PMI randomized controlled study in confinement showed significant reductions in biomarkers of exposure to HPHCs by 47% to 96% in smokers who were switched to IQOS for five days with equivalent nicotine uptake from IQOS compared to participants’ brands of cigarettes^80^. Similar studies for menthol IQOS in Japan and the US showed 50–94% reductions in biomarkers of exposure to HPHCs^81,82^. Other studies switching smokers to IQOS resulted in significant reductions in biomarkers of exposure to TSNAs (about 56%), carbon monoxide (about 77%), benzene (about 94%), 1,3-butadiene (about 92%), and acrolein (about 58%)^83-85^. However, a multicenter ambulatory trial for 26 weeks in the US, reported more modest reductions (16–49%) in biomarkers of HPHCs, which were attributed to the study design. This study showed even fewer reductions (about 10%) among dual users of IQOS and CS^86^. In terms of nicotine delivery from IQOS, a randomized crossover study showed that the nicotine delivery rate was similar between IQOS and CS with lower plasma nicotine peak after IQOS use (70% of CS peak)^87^, and another study reported a similar pharmacokinetic profile of nicotine from IQOS and CS with similar user satisfaction^88^. Estimation of lifetime cancer and non-cancer risks from 8 HTPs (including IQOS) compared to 273 cigarette brands showed that cancer risk decreased by more than one order of magnitude and a significantly higher margin of exposure (MOE) for non-cancer risks^89^.

An independent study showed that IQOS use, like e-cigarettes, led to lower level of end tidal carbon monoxide (eCO) compared to CS among current smokers. However, the authors expressed concern about the longer term effect of eCO increase from baseline after IQOS and e-cigarette use^90^. Another study showed a small but reliable increase in eCO after an IQOS use session^91^. A third study showed no increase in eCO post-IQOS use sessions^92^. A chronic study showed that smokers who switched to IQOS for six months had significantly lower eCO, within the range of non-smokers^93^.

Independent research assessed the MOE to toxic emissions from IQOS compared to CS. It showed higher individual MOEs for all compounds in IQOS emissions (less risk) and 23 times higher combined MOE for all toxic compounds (excluding nicotine) than CS^94^. Also, a study estimated the carcinogenic potency of secondhand smoke from IQOS to be three orders of magnitude lower than cigarettes^95^, and another study showed that IQOS does not impair indoor air quality and does not lead to acute health risks for bystanders^96^.

A report from a competing manufacturer on a randomized controlled trial, Glo and IQOS reduced urinary biomarkers of exposure (i.e. tobacco-specific nitrosamines, carbonyls, VOCs, and PAHs) by 20–90% in Japanese smokers who switched to these products for five days in confinement^97^.

*Biomarkers of health effects*

PMI researchers reported a controlled clinical study that applied systems pharmacology and showed that exposure-response gene signature in blood was similarly reduced in smoking cessation or switching to IQOS groups compared to continued smoking^98^. Moreover, a meta-analysis of four randomized confinement clinical studies corroborated the same result^99^. The multicenter trial discussed in the biomarkers of exposure section showed statistically significant improvement in high-density lipoprotein cholesterol in serum, white blood cell count in blood, carboxyhemoglobin, forced expiratory volume in one second (FEV1), and total NNAL after switching to IQOS for 6 months in smokers^86^. Another study found that the use of menthol IQOS for 5 days by smokers reduced biomarkers of oxidative stress, platelet activation, white blood cell count, and endothelial function, and better lipid metabolism and lung function^100^. A similar study in the US yielded the same reduction in biomarkers of potential harm^101^.

An independent study evaluated the acute impact of IQOS use on pulmonary function in smokers and non-smokers, showing a significant decrease in measures of airway function (flow, volume, and diffusion capacity) and oxygen saturation and almost a significant increase in eCO and airway resistance^102^. Another study showed that exclusive use of IQOS has minimal effect on mucociliary clearance function, as reflected by saccharin test transit time^103^.

Also, an independent study showed that IQOS or CS exposure by current smokers led to acute arterial stiffness, as reflected by higher brachial and systolic blood pressure^104^. A crossover study of smokers showed that the use of IQOS, e-cigarettes, or CS was associated with acute oxidative stress, platelet function, flow-mediated dilation, and blood pressure, with CS being the most detrimental among the three products^105^. Another study showed that IQOS use similar to CS impaired systolic and diastolic myocardial function among current IQOS users, but unlike CS, had no adverse effect on blood pressure^106^. In contrast, a study showed that IQOS use, like CS and e-cigarette use, increased blood pressure and arterial stiffness, and eCO was elevated for all products for up to 60 min^107^. A study assessed the acute (after a use session) and chronic (after one month of being switched to IQOS use) impact of IQOS and CS on endothelial function, arterial stiffness, myocardial deformation, oxidative stress, and platelet activation among smokers. The data showed that IQOS did not have an acute detrimental effect on markers of vascular function, oxidative stress, and platelet activation, and the results were corroborated by the improvement in endothelial function in the chronic phase of the study. This improvement was attributed to reduced CO exposure or reduced nicotine intake^108^. A study showed that HTP (mainly IQOS) use led to abnormal DNA methylation and gene expression profiles, yet to a lower extent than CS^109^.

A few case studies of hospitalization upon using IQOS were also reported. A 20-year-old man developed acute eosinophilic pneumonia after doubling daily IQOS consumption (from 20 to 40 sticks)^110^. Another case study reported the same observation for a 16-year-old youth who started using IQOS 2 weeks before hospitalization^111^. Similarly, a subacute lung injury of a 56-year-old man using IQOS for 2.5 years was reported^112^. In contrast, a study focusing on health benefits for IQOS users with a history of pulmonary diseases showed that in a small cohort of smokers with COPD who switched to IQOS for three years, there was a substantial decrease in COPD exacerbations and improvements in respiratory symptoms and exercise tolerance^113^.

**Figure 3:**
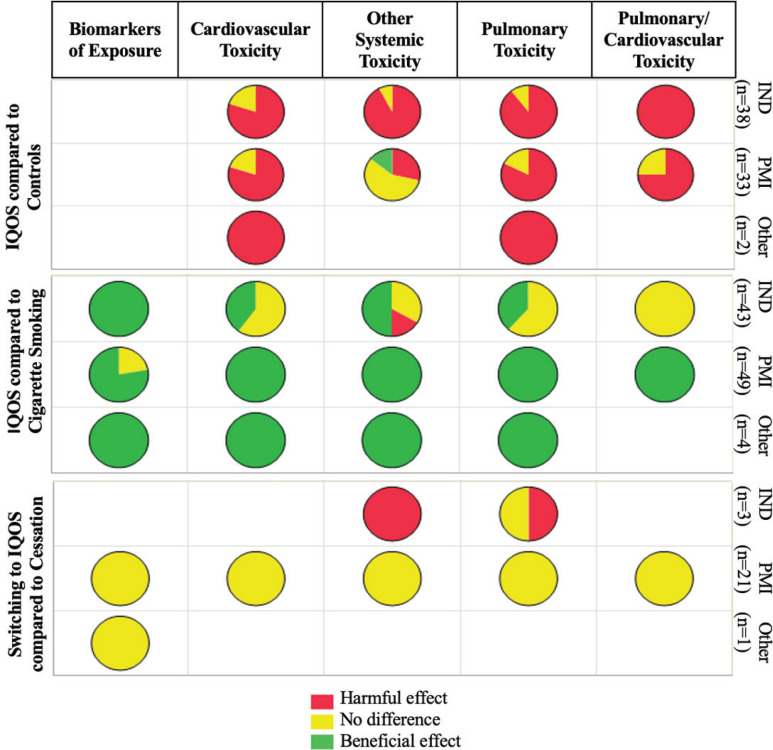
Comparison of toxicity and health impact of IQOS in publications from independent research (IND), PMI, and other HTP manufacturers (Other) to control, cigarette smoking, and smoking cessation conditions. Empty boxes mean that no studies are available in this category

In summary, we compared data from independent research, PMI, and other HTP manufacturers regarding the toxicity and health effects of IQOS. The body systems most studied in independent and PMI research are the pulmonary and cardiovascular systems, yet scattered literature exists on other systems. The use of systems toxicology to generate toxicity data on IQOS was unique to PMI studies, and several initiatives were taken to validate the utility of this approach (Figure 3)^114^. For instance, PMI conducted a crowd-sourcing validation of their systems toxicology approach to assess IQOS toxicity, in which experts recruited through a third party performed modeling on data collected from mice and humans, concluding nearly no harmful effect of IQOS^115^. Moreover, they conducted a peer review to assess the validity of the data generated and the robustness of their systems toxicology approach used in the IQOS MRTP application to the FDA^116,117^. However, no independent research has been conducted on IQOS toxicity using systems toxicology, which is critically needed to provide checks and balances.

Figure 3 summarizes the data comparing the toxicity and health impact of IQOS to controls (exposed to air), cigarette smoking, and smoking cessation models, including *in vitro, in vivo*, and human perspectives. This comparison is focused on the general conclusion of the data reports and does not include a detailed assessment of the methodologies used. Except for one PMI study that showed IQOS exposure has beneficial effects, independent and PMI studies reported harmful or no different effects of IQOS compared to control. All PMI and other HTP manufacturers’ studies reported beneficial effects of IQOS compared to CS. However, the independent evidence was mixed, reporting beneficial, harmful, or similar effects of IQOS compared to CS. PMI and other manufacturers’ data showed an equivalent reduction in toxicity when smokers (or animal models) were switched to IQOS compared to cessation, while some independent research showed harmful effects. It should be noted that our previous systematic review on IQOS content and emissions concluded that industry-supported and independent research agreed on IQOS efficient nicotine delivery and reduced emissions of most cigarette smoking toxicants. Yet, they diverged on increased emissions of chemicals and toxicants in the FDA’s HPHC list or beyond^17^ (Figure 3).

Due to the wide scope of this review, including *in vitro*, *in vivo*, and human studies, the vast literature data were more suitably summarized as a scoping review rather than a systematic review for space constraints. Additionally, the quality of summarized studies and the used methodologies were not evaluated as part of this review of the literature on IQOS toxicity. Nonetheless, this scoping review highlights the general trends in the data on IQOS toxicity and health effects from industry-related and independent researchers. This review aims to emphasize the need for additional independent data on IQOS toxicity and health effects to provide checks and balances, ultimately benefiting all stakeholders, including the product manufacturers. While an updated search could enhance the review, it will not impact its main conclusions.

## CONCLUSION

The ever-growing tobacco product landscape complicates tobacco control, especially as stakeholders, including regulatory authorities, independent scientists, and the tobacco industry, tend to compare the toxicity and health effects of new tobacco products to cigarettes, focusing on the relative rather than the absolute risk of these new products. IQOS is a new tobacco product with extensive data generated by its manufacturers and independent researchers, although to a less extent by the latter. Our comparison of the data from both sources showed that they may not always converge on the reduced risk potential of IQOS compared to cigarettes. There is a need for more data on IQOS, especially on the health effects of long-term use among switching smokers, dual users, as well as novice exclusive users.

## Supplementary Material



## Data Availability

The data supporting this research can be found in the Supplementary file.
