# Supplementary material for: A scoping review of the toxicity and health impact of IQOS
Source: Tob Induc Dis. 2024 Jun 3;22:10.18332/tid/188867. doi: 10.18332/tid/188867 (PMC11145630; doi:10.18332/tid/188867)
Supplement: Supplementary file 1 [file TID-22-97-s1.pdf]

# Supplementary Material

**Table S1.** Collection of the 103 articles included in this narrative review on IQOS toxicity and health impact. The list is sorted according to the topic, data source, year of publication, toxicity/health effects, and study design.

| Title | DOI/PMID/URL | Data Source | Topic | Year | Toxicity/Health effects | Study Design |
|---|---|---|---|---|---|---|
| 3-D Nasal Cultures: Systems Toxicological Assessment of a Candidate Modified-Risk Tobacco Product | 10.14573/altex.1605041 | PMI | Tox | 2017 | Pulmonary toxicity | Systems Toxicology |
| A six-month systems toxicology inhalation/cessation study in ApoE(- / -) mice to investigate cardiovascular and respiratory exposure effects of modified risk tobacco products, CHTP 1.2 and THS 2.2, compared with conventional cigarettes | 10.1016/j.fct.2019.02.008 | PMI | Tox | 2019 | Pulmonary/cardiovascular toxicity | Systems Toxicology |
| A systems toxicology approach for comparative assessment: Biological impact of an aerosol from a candidate modified-risk tobacco product and cigarette smoke on human organotypic bronchial epithelial cultures | 10.1016/j.tiv.2016.11.009 | PMI | Tox | 2017 | Pulmonary toxicity | Systems Toxicology |
| Aerosol from a candidate modified risk tobacco product has reduced | 10.1016/j.fct.2015.09.016 | PMI | Tox | 2015 | Cardiovascular Toxicity | In Vitro |

| | | | | | | |
|---|---|---|---|---|---|---|
| effects on chemotaxis and transendothelial migration compared to combustion of conventional cigarettes | | | | | | |
| Aerosol from Tobacco Heating System 2.2 has reduced impact on mouse heart gene expression compared with cigarette smoke | 10.1016/j.fct.2017.01.013 | PMI | Tox | 2017 | Cardiovascular Toxicity | Systems Toxicology |
| An 8-Month Systems Toxicology Inhalation/Cessation Study in Apoe-/- Mice to Investigate Cardiovascular and Respiratory Exposure Effects of a Candidate Modified Risk Tobacco Product, THS 2.2, Compared With Conventional Cigarettes | 10.1093/toxsci/kfv243 | PMI | Tox | 2016 | Pulmonary/cardiovascular toxicity | Systems Toxicology |
| Comparative effects of a candidate modified-risk tobacco product Aerosol and cigarette smoke on human organotypic small airway cultures: a systems toxicology approach | 10.1039/c7tx00152e | PMI | Tox | 2017 | Pulmonary toxicity | Systems Toxicology |
| Comparative systems toxicology analysis of cigarette smoke and aerosol from a candidate modified risk tobacco product in organotypic human gingival epithelial cultures: A 3-day repeated exposure study | 10.1016/j.fct.2016.12.027 | PMI | Tox | 2017 | Other Systemic Toxicity | Systems Toxicology |

| | | | | | | |
|---|---|---|---|---|---|---|
| Comparison of monoamine oxidase inhibition by cigarettes and modified risk tobacco products | 10.1016/j.toxrep.2019.11.008 | PMI | Tox | 2019 | Other Systemic Toxicity | In Vitro |
| Crowd-Sourced Verification of Computational Methods and Data in Systems Toxicology: A Case Study with a Heat-Not-Burn Candidate Modified Risk Tobacco Product | 10.1021/acs.chemrestox.6b00345 | PMI | Tox | 2017 | blood transcriptomics | Systems Toxicology |
| Effects of cigarette smoke, cessation and switching to a candidate modified risk tobacco product on the liver in Apoe(-/-) mice - a systems toxicology analysis | 10.3109/08958378.2016.1150368 | PMI | Tox | 2016 | Other Systemic Toxicity | Systems Toxicology |
| Effects of Cigarette Smoke, Cessation, and Switching to Two Heat-Not-Burn Tobacco Products on Lung Lipid Metabolism in C57BL/6 and Apoe-/- Mice-An Integrative Systems Toxicology Analysis | 10.1093/toxsci/kfv244 | PMI | Tox | 2016 | Pulmonary toxicity | Systems Toxicology |
| Evaluation of the Tobacco Heating System 2.2 (THS2.2). Part 5: microRNA expression from a 90-day rat inhalation study indicates that exposure to THS2.2 aerosol causes reduced effects on lung tissue compared with cigarette smoke | 10.1016/j.yrtph.2016.11.018 | PMI | Tox | 2016 | Pulmonary toxicity | Systems Toxicology |

| | | | | | | |
|---|---|---|---|---|---|---|
| Evaluation of the Tobacco Heating System 2.2. Part 4: 90-day OECD 413 rat inhalation study with systems toxicology endpoints demonstrates reduced exposure effects compared with cigarette smoke | 10.1016/j.yrtph.2016.10.015 | PMI | Tox | 2016 | Pulmonary toxicity | systems Toxicology |
| Evaluation of the Tobacco Heating System 2.2. Part 6: 90-day OECD 413 rat inhalation study with systems toxicology endpoints demonstrates reduced exposure effects of a mentholated version compared with mentholated and non-mentholated cigarette smoke | 10.1016/j.yrtph.2016.11.004 | PMI | Tox | 2016 | Pulmonary toxicity | Systems Toxicology |
| Evaluation of the Tobacco Heating System 2.2. Part 7: Systems toxicological assessment of a mentholated version revealed reduced cellular and molecular exposure effects compared with mentholated and non-mentholated cigarette smoke | 10.1016/j.yrtph.2016.11.001 | PMI | Tox | 2016 | Pulmonary toxicity | Systems Toxicology |
| Impact of switching to a heat-not-burn tobacco product on CYP1A2 activity | 10.1016/j.toxrep.2020.10.017 | PMI | Tox | 2020 | Other Systemic Toxicity | In Vivo |
| In Vitro Systems Toxicology Assessment of a Candidate Modified Risk Tobacco Product Shows Reduced Toxicity | 10.1021/acs.chemrestox.5b00321 | PMI | Tox | 2016 | Pulmonary toxicity | Systems Toxicology |

| | | | | | | |
|---|---|---|---|---|---|---|
| Compared to That of a Conventional Cigarette | | | | | | |
| Mitochondrial Network and Biogenesis in Response to Short and Long-Term Exposure of Human BEAS-2B Cells to Aerosol Extracts from the Tobacco Heating System 2.2 | 10.33594/000000216 | PMI | Tox | 2020 | Pulmonary toxicity | In Vitro |
| Impact of 6-Month Exposure to Aerosols From Potential Modified Risk Tobacco Products Relative to Cigarette Smoke on the Rodent Gastrointestinal Tract | 10.3389/fmicb.2021.587745 | PMI | Tox | 2021 | Other Systemic Toxicity | In Vivo |
| Multi-omics systems toxicology study of mouse lung assessing the effects of aerosols from two heat-not-burn tobacco products and cigarette smoke | 10.1016/j.csbj.2020.04.011 | PMI | Tox | 2020 | Pulmonary toxicity | systems Toxicology |
| Reduced Chronic Toxicity and Carcinogenicity in A/J Mice in Response to Life-Time Exposure to Aerosol from a Heated Tobacco Product Compared with Cigarette Smoke | 10.1093/toxsci/kfaa131 | PMI | Tox | 2020 | Pulmonary toxicity | In Vivo |
| Respiratory effects of exposure to aerosol from the candidate modified-risk tobacco product THS 2.2 in an 18-month systems toxicology study with A/J mice | 10.1093/toxsci/kfaa132 | PMI | Tox | 2020 | Pulmonary toxicity | Systems Toxicology |

| | | | | | | |
|---|---|---|---|---|---|---|
| Structural, functional, and molecular impact on the cardiovascular system in ApoE(-/-) mice exposed to aerosol from candidate modified risk tobacco products, Carbon Heated Tobacco Product 1.2 and Tobacco Heating System 2.2, compared with cigarette smoke | 10.1016/j.cbi.2019.108887 | PMI | Tox | 2020 | Cardiovascular Toxicity | Systems Toxicology |
| Systems Toxicology Assessment of the Biological Impact of a Candidate Modified Risk Tobacco Product on Human Organotypic Oral Epithelial Cultures | 10.1021/acs.chemrestox.6b00174 | PMI | Tox | 2016 | Other Systemic Toxicity | Systems Toxicology |
| Systems toxicology meta-analysis of in vitro assessment studies: biological impact of a candidate modified-risk tobacco product aerosol compared with cigarette smoke on human organotypic cultures of the aerodigestive tract | 10.1039/c7tx00047b | PMI | Tox | 2017 | Pulmonary toxicity | Systems Toxicology |
| Systems toxicology study reveals reduced impact of heated tobacco product aerosol extract relative to cigarette smoke on premature aging and exacerbation effects in aged aortic cells in vitro | 10.1007/s00204-021-03123-y | PMI | Tox | 2021 | Cardiovascular Toxicity | Systems Toxicology |
| Systems toxicology-based assessment of the candidate modified risk tobacco product THS2.2 for the adhesion of | 10.1016/j.tox.2015.11.007 | PMI | Tox | 2016 | Cardiovascular Toxicity | Systems Toxicology |

| | | | | | | |
|---|---|---|---|---|---|---|
| monocytic cells to human coronary arterial endothelial cells | | | | | | |
| The biological effects of long-term exposure of human bronchial epithelial cells to total particulate matter from a candidate modified-risk tobacco product | 10.1016/j.tiv.2018.02.019 | PMI | Tox | 2018 | Pulmonary toxicity | Systems Toxicology |
| Tobacco Heating System 2.2 has a limited impact on DNA methylation of candidate enhancers in mouse lung compared with cigarette smoke | 10.1016/j.fct.2018.11.020 | PMI | Tox | 2019 | Pulmonary toxicity | In Vivo |
| Toxicological assessment of Tobacco Heating System 2.2: Findings from an independent peer review | 10.1016/j.yrtph.2019.03.007 | PMI | Tox | 2019 | Pulmonary/cardiovascular toxicity | Systems Toxicology |
| Impact of aerosols on liver xenobiotic metabolism: A comparison of two methods of exposure. | https://doi.org/10.1016/j.tiv.2021.105277 | PMI | Tox | 2022 | Pulmonary toxicity | In Vitro |
| Comparing the preclinical risk profile of inhalable candidate and potential candidate modified risk tobacco products: A bridging use case | 10.1016/j.toxrep.2020.09.004 | PMI | Tox | 2020 | Pulmonary/cardiovascular toxicity | Systems Toxicology |
| "Assessment of mitochondrial function following short- and long-term exposure of human bronchial epithelial cells to total particulate matter from a candidate modified-risk | https://doi.org/10.1016/j.fct.2018.02.013 | PMI | Tox | 2018 | Pulmonary toxicity | In Vitro |

| | | | | | | |
|---|---|---|---|---|---|---|
| tobacco product and reference cigarettes" | | | | | | |
| Effects of cigarette smoke and tobacco heating aerosol on color stability of dental enamel, dentin, and composite resin restorations | 10.3290/j.qi.a41601 | PMI | Tox | 2019 | Other Systemic Toxicity | In Vitro |
| A Meta-Analysis of the Performance of a Blood-Based Exposure Response Gene Signature Across Clinical Studies on the Tobacco Heating System 2.2 (THS 2.2) | 10.3389/fphar.2019.00198 | PMI | Health | 2019 | Other Systemic Toxicity | Clinical |
| Assessment of the reduction in levels of exposure to harmful and potentially harmful constituents in Japanese subjects using a novel tobacco heating system compared with conventional cigarettes and smoking abstinence: A randomized controlled study in confinement | 10.1016/j.yrtph.2016.09.014 | PMI | Health | 2016 | Biomarkers of Exposure | Clinical |
| Biomarker of exposure level data set in smokers switching from conventional cigarettes to Tobacco Heating System 2.2, continuing smoking or abstaining from smoking for 5 days | 10.1016/j.dib.2016.11.047 | PMI | Health | 2017 | Biomarkers of Exposure | Clinical |
| Comparison of the Pharmacokinetics of Nicotine Following Single and Ad Libitum Use of a Tobacco Heating | 10.1093/ntr/ntv220 | PMI | Health | 2016 | Biomarkers of Exposure | Clinical |

| | | | | | | |
|---|---|---|---|---|---|---|
| System or Combustible Cigarettes | | | | | | |
| Effects of Switching to a Heat-Not-Burn Tobacco Product on Biologically Relevant Biomarkers to Assess a Candidate Modified Risk Tobacco Product: A Randomized Trial | 10.1158/1055-9965.epi-18-0915 | PMI | Health | 2019 | Biomarkers of Exposure/health effects | Clinical |
| Effects of Switching to the Menthol Tobacco Heating System 2.2, Smoking Abstinence, or Continued Cigarette Smoking on Clinically Relevant Risk Markers: A Randomized, Controlled, Open-Label, Multicenter Study in Sequential Confinement and Ambulatory Settings (Part 2) | 10.1093/ntr/ntx028 | PMI | Health | 2018 | Pulmonary/cardiovascular toxicity | Clinical |
| Effects of Switching to the Tobacco Heating System 2.2 Menthol, Smoking Abstinence, or Continued Cigarette Smoking on Biomarkers of Exposure: A Randomized, Controlled, Open-Label, Multicenter Study in Sequential Confinement and Ambulatory Settings (Part 1) | 10.1093/ntr/ntw287 | PMI | Health | 2018 | Biomarkers of Exposure | Clinical |
| Evaluation of the Tobacco Heating System 2.2. Part 8: 5-Day randomized reduced | 10.1016/j.yrtph.2016.11.003 | PMI | Health | 2016 | Biomarkers of Exposure | Clinical |

| | | | | | | |
|---|---|---|---|---|---|---|
| exposure clinical study in Poland | | | | | | |
| Evaluation of the tobacco heating system 2.2. Part 9: Application of systems pharmacology to identify exposure response markers in peripheral blood of smokers switching to THS2.2 | 10.1016/j.yrtph.2016.11.011 | PMI | Health | 2016 | Other Systemic Toxicity | Clinical |
| Favorable Changes in Biomarkers of Potential Harm to Reduce the Adverse Health Effects of Smoking in Smokers Switching to the Menthol Tobacco Heating System 2.2 for 3 Months (Part 2) | 10.1093/ntr/ntz084 | PMI | Health | 2020 | Cardiovascular Toxicity | Clinical |
| Nicotine pharmacokinetic profiles of the Tobacco Heating System 2.2, cigarettes and nicotine gum in Japanese smokers | 10.1016/j.yrtph.2017.07.032 | PMI | Health | 2017 | Biomarkers of Exposure | Clinical |
| Reduced Exposure to Harmful and Potentially Harmful Smoke Constituents With the Tobacco Heating System 2.1 | 10.1093/ntr/ntw164 | PMI | Health | 2017 | Biomarkers of Exposure | Clinical |
| Reduction in Exposure to Selected Harmful and Potentially Harmful Constituents Approaching Those Observed Upon Smoking Abstinence in Smokers Switching to the Menthol Tobacco Heating System 2.2 for 3 Months (Part 1) | 10.1093/ntr/ntz013 | PMI | Health | 2020 | Biomarkers of Exposure | Clinical |
| Cancer potencies and margin of exposure used | 10.1007/s00204-020-02924-x | PMI | Health | 2021 | Biomarkers of Exposure | Risk assessment |

| | | | | | | |
|---|---|---|---|---|---|---|
| for comparative risk assessment of heated tobacco products and electronic cigarettes aerosols with cigarette smoke | | | | | | |
| Comparative study of the effects of cigarette smoke versus next generation tobacco and nicotine product extracts on endothelial function | 10.1016/j.redox.2021.102150 | Other | Tox | 2021 | Cardiovascular Toxicity | In Vitro |
| In vitro RNA-seq-based toxicogenomics assessment shows reduced biological efect of tobacco heating products when compared to cigarette smoke | 10.1038/s41598-018-19627-0 | Other | Tox | 2018 | Pulmonary toxicity | Systems Toxicology |
| The use of human induced pluripotent stem cells to screen for developmental toxicity potential indicates reduced potential for non-combusted products, when compared to cigarettes | 10.1016/j.crtox.2020.11.001 | Other | Tox | 2020 | Other Systemic Toxicity | In Vitro |
| Changes in Biomarkers of Exposure on Switching From a Conventional Cigarette to Tobacco Heating Products: A Randomized, Controlled Study in Healthy Japanese Subjects | 10.1093/ntr/nty104 | Other | Health | 2019 | Biomarkers of Exposure | Clinical |
| Acute Effects of Heated Tobacco Product (IQOS) Aerosol Inhalation on Lung Tissue Damage and | 10.1093/ntr/ntaa267 | Ind | Tox | 2021 | Pulmonary toxicity | In Vivo |

| | | | | | | |
|---|---|---|---|---|---|---|
| Inflammatory Changes in the Lungs | | | | | | |
| Assessment of tobacco heating system 2.4 on osteogenic differentiation of mesenchymal stem cells and primary human osteoblasts compared to conventional cigarettes | 10.4252/WJSC.V12.I8.841 | Ind | Tox | 2020 | Other Systemic Toxicity | In Vitro |
| Cigarette smoke extract and heated tobacco products promote ferritin cleavage and iron accumulation in human corneal epithelial cells | 10.1038/s41598-021-97956-3 | Ind | Tox | 2021 | Other Systemic Toxicity | In Vitro |
| Comparison of cytotoxicity of cigarette smoke extract derived from heat-not-burn and combustion cigarettes in human vascular endothelial cells | 10.1016/j.jphs.2021.07.005 | Ind | Tox | 2021 | Cardiovascular Toxicity | In Vitro |
| Comparison of cytotoxicity of IQOS aerosols to smoke from Marlboro Red and 3R4F reference cigarettes | 10.1016/j.tiv.2019.104652 | Ind | Tox | 2019 | Pulmonary toxicity | In Vitro |
| Cytotoxic effects of heated tobacco products (HTP) on human bronchial epithelial cells | 10.1136/tobaccocontrol-2018-054317 | Ind | Tox | 2018 | Pulmonary toxicity | In Vitro |
| Effects of conventional and heated tobacco product smoking on discoloration of artificial denture teeth | 10.1016/j.prosdent.2020.05.031 | Ind | Tox | 2021 | Other Systemic Toxicity | In Vitro |
| Effects of Exposure to Tobacco Cigarette, Electronic Cigarette and Heated Tobacco Product on Adipocyte Survival | 10.3390/toxics8010009 | Ind | Tox | 2020 | Other Systemic Toxicity | In Vitro |

| | | | | | | |
|---|---|---|---|---|---|---|
| and Differentiation In Vitro | | | | | | |
| Effects of Fetal Exposure to Heat-Not-Burn Tobacco on Testicular Function in Male Offspring | 10.1248/bpb.b20-00390 | Ind | Tox | 2020 | Other Systemic Toxicity | In Vivo |
| Heat-Not-Burn cigarette induces oxidative stress response in primary rat alveolar epithelial cells | 10.1371/journal.pone.0242789 | Ind | Tox | 2020 | Pulmonary toxicity | In Vitro |
| Heat-not-burn tobacco (IQOS), oral fibroblasts and keratinocytes: cytotoxicity, morphological analysis, apoptosis and cellular cycle. An in vitro study | 10.1111/jre.12888 | Ind | Tox | 2021 | Other Systemic Toxicity | In Vitro |
| Heat-Not-Burn Tobacco Products: The Devil in Disguise or a Considerable Risk Reduction? | 10.7895/ijadr.250+[@[Type of paper]] | Ind | Tox | 2018 | Biomarkers of Exposure | Risk assessment |
| Immunotoxic mechanisms of cigarette smoke and heat-not-burn tobacco vapor on Jurkat T cell functions | 10.1016/j.envpol.2020.115863 | Ind | Tox | 2021 | Other Systemic Toxicity | In Vitro |
| Role of diabetes in lung injury from acute exposure to electronic cigarette, heated tobacco product, and combustible cigarette aerosols in an animal model | 10.1371/journal.pone.0255876 | Ind | Tox | 2021 | Pulmonary toxicity | In Vivo |
| Toxic mechanisms of cigarette smoke and heat-not-burn tobacco vapor inhalation on rheumatoid arthritis | 10.1016/j.scitotenv.2021.151097 | Ind | Tox | 2021 | Other Systemic Toxicity | In Vivo/in vitro |

| | | | | | | |
|---|---|---|---|---|---|---|
| Unburned Tobacco Cigarette Smoke Alters Rat Ultrastructural Lung Airways and DNA | 10.1093/ntr/ntab108 | Ind | Tox | 2021 | Pulmonary toxicity | In Vivo |
| Vascular endothelial function is impaired by aerosol from a single IQOS HeatStick to the same extent as by cigarette smoke | 10.1136/tobaccocontrol-2018-054325 | Ind | Tox | 2018 | Cardiovascular Toxicity | In Vivo |
| Different Effects of Cigarette Smoke, Heated Tobacco Product and E-Cigarette Vapour on Orbital Fibroblasts in Graves' Orbitopathy; a Study by Real Time Cell Electronic Sensing. | https://doi.org/10.3390/molecules27093001 | IND | Tox | 2022 | Other Systemic Toxicity | In Vitro |
| A Newly Developed Aerosol Exposure Apparatus for Heated Tobacco Products for In Vivo Experiments Can Deliver Both Particles and Gas Phase With High Recovery and Depicts the Time-Dependent Variation in Nicotine Metabolites in Mouse Urine | | Ind | Tox | | | |
| Comparable Impairment of Vascular Endothelial Function by a Wide Range of Electronic Nicotine Delivery Devices | https://doi.org/10.1093/ntr/ntac019 | IND | Tox | 2022 | Cardiovascular Toxicity | In Vivo |
| Exposure to the heated tobacco product IQOS generates apoptosis-mediated pulmonary | https://doi.org/10.1152/ajplung.00215.2021 | IND | Tox | 2022 | Pulmonary toxicity | In Vivo |

| | | | | | | |
|---|---|---|---|---|---|---|
| emphysema in murine lungs | | | | | | |
| Exposure to aerosol extract from heated tobacco products causes a drastic decrease of glutathione and protein carbonylation in human lung epithelial cells. | https://doi.org/10.1016/j.bbrc.2021.12.004 | IND | Tox | 2021 | Pulmonary toxicity | In Vitro |
| Heated Tobacco Products Impair Cell Viability, Osteoblastic Differentiation, and Bone Fracture-Healing | | Ind | Tox | 2021 | Other Systemic Toxicity | In Vitro |
| Increased oxidative stress and effects on inflammatory cytokine secretion by heated tobacco products aerosol exposure to mice. | https://doi.org/10.1016/j.bbrc.2022.04.042 | IND | Tox | 2022 | Pulmonary toxicity | In Vivo |
| Chronic exposure to IQOS results in impaired pulmonary function and lung tissue damage in mice | https://doi.org/10.1016/j.toxlet.2022.11.022 | Ind | Tox | 2023 | Pulmonary toxicity | In Vivo |
| Exposure to Heated Tobacco Products Aerosol Causes Acute Stress Responses in the Lung of Mouse | https://doi.org/10.3390/antiox11122329 | Ind | Tox | 2022 | Pulmonary toxicity | In Vivo |
| Acute effect of heat-not-burn versus standard cigarette smoking on arterial stiffness and wave reflections in young smokers | 10.1177/2047487320918365 | Ind | Health | 2021 | Cardiovascular Toxicity | Clinical |
| Acute Effects of a Heat-Not-Burn Tobacco Product on Pulmonary Function | 10.3390/medicina56060292 | Ind | Health | 2020 | Pulmonary toxicity | Clinical |

| | | | | | | |
|---|---|---|---|---|---|---|
| Acute Effects of Heat-Not-Burn, Electronic Vaping, and Traditional Tobacco Combustion Cigarettes: The Sapienza University of Rome-Vascular Assessment of Proatherosclerotic Effects of Smoking (SUR-VAPES) 2 Randomized Trial | 10.1161/jaha.118.010455 | Ind | Health | 2019 | Cardiovascular Toxicity | Clinical |
| Acute effects of JUUL and IQOS in cigarette smokers | 10.1136/tobaccocontrol-2019-055475 | Ind | Health | 2020 | Biomarkers of Health Effects | Clinical |
| Acute eosinophilic pneumonia following heat-not-burn cigarette smoking | 10.1002/rcr2.190 | Ind | Health | 2016 | Pulmonary toxicity | Case Study |
| Assessment of industry data on pulmonary and immunosuppressive effects of IQOS | 10.1136/tobaccocontrol-2018-054296 | Ind | Health | 2018 | Pulmonary toxicity | clinical/in vivo |
| Comparison of End Tidal Carbon Monoxide Levels between Conventional Cigarette, Electronic Cigarette and Heated Tobacco Product among Asiatic Smokers | 10.1080/10826084.2020.1781180 | Ind | Health | 2020 | Biomarkers of Exposure | Clinical |
| Comparison of IQOS (heated tobacco) and cigarette smoking on cardiac functions by two-dimensional speckle tracking echocardiography | 10.1016/j.taap.2021.115575 | Ind | Health | 2021 | Cardiovascular Toxicity | Clinical |
| Criminal mercury vapor poisoning using heated tobacco product | 10.1007/s00414-018-1923-4 | Ind | Health | 2019 | | |
| Differential effects of heat-not-burn and | 10.1038/s41598-021-91245-9 | Ind | Health | 2021 | Cardiovascular Toxicity | Clinical |

| | | | | | | |
|---|---|---|---|---|---|---|
| conventional cigarettes on coronary flow, myocardial and vascular function | | | | | | |
| DNA methylation abnormalities and altered whole transcriptome profiles after switching from combustible tobacco smoking to heated tobacco products | 10.1158/1055-9965.Epi-21-0444 | Ind | Health | 2021 | Other Systemic Toxicity | Clinical |
| Estimating the Carcinogenic Potency of Second-Hand Smoke and Aerosol from Cigarettes and Heated Tobacco Products | 10.3390/ijerph17228319 | Ind | Health | 2020 | Biomarkers of Exposure | Clinical |
| Exhaled Carbon Monoxide Levels in Forty Resistant to Cessation Male Smokers after Six Months of Full Switch to Electronic Cigarettes (e-Cigs) or to A Tobacco Heating Systems (THS) | 10.3390/ijerph16203916 | Ind | Health | 2019 | Biomarkers of Exposure | Clinical |
| Health outcomes in COPD smokers using heated tobacco products: a 3-year follow-up | 10.1007/s11739-021-02674-3 | Ind | Health | 2021 | Pulmonary toxicity | Clinical |
| Heat-not-burn cigarettes induce fulminant acute eosinophilic pneumonia requiring extracorporeal membrane oxygenation | 10.1016/j.rmcr.2018.12.002 | Ind | Health | 2019 | Pulmonary toxicity | Case Study |
| Impact of exclusive e-cigarettes and heated tobacco products use on muco-ciliary clearance | 10.1177/20406223211035267 | Ind | Health | 2021 | Pulmonary toxicity | Clinical |
| IQOS(TM) vs. e-Cigarette vs. Tobacco | 10.3390/ijerph15122902 | Ind | Health | 2018 | Biomarkers of Exposure | Clinical |

| | | | | | | |
|---|---|---|---|---|---|---|
| Cigarette: A Direct Comparison of Short-Term Effects after Overnight-Abstinence | | | | | | |
| Modelling the impact of a new tobacco product: review of Philip Morris International's Population Health Impact Model as applied to the IQOS heated tobacco product | 10.1136/tobaccocontrol-2018-054572 | Ind | Health | 2018 | | |
| PMI's own in vivo clinical data on biomarkers of potential harm in Americans show that IQOS is not detectably different from conventional cigarettes. | 10.1136/tobaccocontrol-2018-054413 | Ind | Health | 2018 | Pulmonary/cardiovascular toxicity | Clinical |
| Profiling the Acute Effects of Modified Risk Products: Evidence from the SUR-VAPES (Sapienza University of Rome-Vascular Assessment of Proatherosclerotic Effects of Smoking) Cluster Study | 10.1007/s11883-020-0824-4 | Ind | Health | 2020 | Cardiovascular Toxicity | Clinical |
| Subacute lung injury associated with heated tobacco products | 10.18678/dtfd.896093 | Ind | Health | 2021 | Biomarkers of Health Effects | Case Study |
| The impact of heated tobacco products on arterial stiffness | 10.1177/1358863x20943292 | Ind | Health | 2020 | Cardiovascular Toxicity | Clinical |
| JUUL™ing and Heating Lead to a Worsening of Arterial Stiffness | https://doi.org/10.3390/medicines9040028 | IND | Health | 2022 | Cardiovascular Toxicity | Clinical |
| Assessing acute inhalation health risk caused by exposure to products created by | https://doi.org/10.21668/health.risk/2021.2.06.eng | IND | Health | 2021 | Biomarkers of Exposure | Risk assessment |

| | | | | | | |
|---|---|---|---|---|---|---|
| nicotine-containing stuff consumption in enclosed spaces | | | | | | |

© 2024 Ghazi S. et al.
